# Assessing the Effects on Health Inequalities of Differential Exposure and Differential Susceptibility of Air Pollution and Environmental Noise in Barcelona, 2007–2014

**DOI:** 10.3390/ijerph16183470

**Published:** 2019-09-18

**Authors:** Marc Saez, Guillem López-Casasnovas

**Affiliations:** 1 Research Group on Statistics, Econometrics and Health (GRECS), University of Girona, 17003 Girona, Spain; 2 CIBER of Epidemiology and Public Health (CIBERESP), 28029 Madrid, Spain; 3 Center for Research in Health and Economics (CRES), Universitat Pompeu Fabra, 08005 Barcelona, Spain; guillem.lopez@upf.edu; 4 Department of Economics and Business, Universitat Pompeu Fabra, 08005 Barcelona, Spain; 5 Barcelona Graduate School (BGSE), Universitat Pompeu Fabra, 08005 Barcelona, Spain

**Keywords:** exposure differential, susceptibility differential, ecological regression, spatial misalignment, spatio-temporal adjustment

## Abstract

The hypotheses we intended to contrast were, first, that the most deprived neighborhoods in Barcelona, Spain, present high exposure to environmental hazards (differential exposure) and, secondly, that the health effects of this greater exposure were higher in the most deprived neighborhoods (differential susceptibility). The population studied corresponded to the individuals residing in the neighborhoods of Barcelona in the period 2007–2014. We specified the association between the relative risk of death and environmental hazards and socioeconomic indicators by means of spatio-temporal ecological regressions, formulated as a generalized linear mixed model with Poisson responses. There was a differential exposure (higher in more deprived neighborhoods) in almost all the air pollutants considered, when taken individually. The exposure was higher in the most affluent in the cases of environmental noise. Nevertheless, for both men and women, the risk of dying due to environmental hazards in a very affluent neighborhood is about 30% lower than in a very depressed neighborhood. The effect of environmental hazards was more harmful to the residents of Barcelona’s most deprived neighborhoods. This increased susceptibility cannot be attributed to a single problem but rather to a set of environmental hazards that, overall, a neighborhood may present.

## 1. Introduction

Today, there is abundant evidence that health inequalities exist [1]. Despite this having already been established in the seminal Black Report [2], it was the Acheson Report (Independent Inquiry into Inequalities in Health) that firmly concluded that there is scientific evidence of health inequalities having a socioeconomic explanation [3]. Nowadays, twenty years later, those relationships have mostly been proven [1,4,5,6], with a not insignificant proportion of them being caused by environmental problems [7]. These factors are usually, although not uniquely, linked to socioeconomic conditions [7,8,9,10,11,12]. 

Very related, in the early 1980s, the concept of environmental justice appeared in the United States [13,14]. Some authors have pioneered environmental justice studies in Europe, drawing on frameworks and methods developed in the context of environmental justice in the United States [15,16,17,18,19].

In general, environmental conditions can contribute to socioeconomic inequalities in health in two ways, i.e., independently or, more likely, together [7,9,12,20]. The first is differential exposure: the most economically disadvantaged groups present high exposure to environmental hazards, including, but not limited to, air pollution, while the second is differential susceptibility to exposure: having the major adverse health effects, resulting from environmental problems, among the most economically disadvantaged individuals, due to their greater vulnerability. 

In this article, we are interested in assessing how both concepts lead to the breach in the principle of environmental equity. We search for the exposure and the susceptibility differentials in health that result from the environmental hazards in the city of Barcelona, Spain, during 2007–2014. We adopt an ecological perspective [1], following the conventional approach to spatial epidemiology. We use the neighborhoods of Barcelona as our units of analysis. While the ultimate reason for this decision was the non-availability of data at the individual level, we proceeded with this approach because of the existing broad consensus that not only are the variables at the individual level, but also the area of residence of the individual is the actual socioeconomic determinant of their health [21,22,23]. Thus, the hypotheses we intend to contrast is, first, that the most deprived neighborhoods in the city present high exposure to environmental hazards (differential exposure) and, secondly, that the health effects of this greater exposure are higher in the most deprived neighborhoods (differential susceptibility).

The health effects that we focus on are total mortality rates, stratified by gender. We considered not only air pollution as an environmental problem, but also environmental noise. For instance, in a large city, the single consequence of traffic is not air pollution, as traffic also contributes to 80% of the city’s environmental noise [24]. Although some authors question whether it is air pollution, and not noise, which is associated with adverse health effects [25,26,27], several studies have shown an independent association for both air pollution and environmental noise on adverse health events [28,29,30]. 

The existing literature, mainly from North America and Europe, shows mostly, but not unanimously [7,31,32,33], that the poorest individuals are more exposed to environmental problems, especially to higher levels of air pollution. As regards to environmental noise, and despite less scientific evidence here, the existence of differential exposure, higher for economically disadvantaged individuals, has been demonstrated by some new papers [31,34,35,36]. Others, however, find that it is not the poor but the intermediate groups (neither very rich nor very poor), who are exposed to such traffic-related environmental hazards [37,38]. 

For the hypothesis of differential susceptibility, the general pattern of the existing evidence, and in this case almost unanimous, is that regardless of the level of exposure to air pollutants, it is the poorest who experience the worst health effects [7,33]. However, there is no evidence of a differential susceptibility in the case of environmental noise.

In this paper, we intend to confirm the effect environmental problems have on socioeconomic inequalities in health by using intra-urban geographical areas as the units of analysis [23,39,40,41,42,43,44], given that these are already mostly clustered by socioeconomic conditions [7,12]. What we add to what is already known, is that we do this by using appropriate statistical methods that consider the spatial design of the data currently used. 

First, we control for the problem of ‘misalignment’. In fact, when using a design for spatial data, it is often the case that the data exposure and the health outcomes have different spatial locations, so they are spatially ‘misaligned’ [45] (this problem is also known as the ‘modifiable areal unit’ or the ‘change of support’ problem [45,46]). Most studies address this problem (although not always explicitly) using a two-stage modelling procedure or ‘plug-in’ approach. In this method, predictions from an exposure model (first stage) are used as covariates in a health model (second stage); this being the model of interest [47]. In very few cases, predictions are obtained from exposure models that explicitly incorporate the spatial structure of the data (i.e., kriging, spatial interpolation, etc.). However, even in these situations, the plug-in approach does not consider the uncertainty in the exposure predictions, leading to a complex form of measurement error, which, if not properly controlled, results in the bias of the estimated health effect [47,48].

Second, we explicitly perform a spatio-temporal adjustment. For this, on the one hand, with spatial data it is necessary to distinguish between two sources of extra variability, ‘spatial dependence’ or clustering (i.e., spatial autocorrelation), and non-spatial heterogeneity (i.e., heteroskedasticity) [49,50]. Furthermore, when the data have a temporal component, as is our case, there is time dependence (i.e., autocorrelation). If the spatio-temporal extra variability (i.e., heterogeneity and both, spatial and temporal dependencies) are not controlled for, not only will the variances of the estimators be wrong, but estimators will be biased and inconsistent [51]. This will be the case when the dependent variable is not continuous (i.e., a counting variable, as the number of total deaths) and then seriously compromising the inferences that might be made. 

## 2. Materials and Methods

### 2.1. Data Setting

We use a small area spatio-temporal ecological design. The population studied corresponded to the individuals residing in the neighbourhoods of Barcelona in the period 2007–2014. Barcelona, capital of Catalonia, Spain, and located on the North Mediterranean coast, 192 km from the French border, is the second city of Spain, after Madrid, in terms of its population and its economic activity. According to the Statistical Institute of Catalonia (IDESCAT), the population of Barcelona (January 1, 2015) was 1,604,555 inhabitants, 759,820 men (47.06%) and 845,035 women (52.94%) [52]. Barcelona is the second most populated city in Spain, after Madrid, and the eleventh most populated in the European Union. The density of population is very high, 15,839.6 hab/km^2^. It is a city with an aging population (21.62% of the population are aged 65 years or more, 18.17% of the men and 24.97% of the women, over total population). For administrative and statistical purposes, the Barcelona City Council has divided the city into 73 neighborhoods [53], and these were used as the units of analysis. In 2014, the median of habitants per neighborhood was 20,184 (9748 men and 10,436 women average with an interquartile range equal to 10,381–31,007), the median of the density of population was 242,288 hab/km^2^ (with an interquartile rank equal to 11,459–350,585 hab/km^2^) [50]. Also in 2014, the neighborhood with fewest inhabitants was ‘La Clota’, with 529 inhabitants (259 men and 270 women), and the neighbourhood with the most inhabitants was ‘La nova Esquerra de l’Eixample’ with 57,863 inhabitants (26,806 men and 31,057 women). The neighbourhood with the least density was ‘La Marina del Prat Vermell’, with 80.60 hab/km^2^ and ‘Sants-Badal’ had the greatest density with 59,134.15 hab/km^2^ [54]. Although the levels of spatial segregation are not as pronounced as those of the countries around us (for example, France), segregation has a structural character in the city of Barcelona, being significantly high since at least 2001 and has come increasing especially since the years of the Great Recession (which began in the first quarter of 2009 in Spain) [55]. The census tracts in which the lowest incomes are grouped are concentrated in the neighborhoods of the Besòs river (which delimits Nord-Nordest city of Barcelona) [55]. These are degraded neighborhoods, mass housing estates built in the 60s and 70s and areas born of marginal urbanization processes [56]. They concentrate the lowest income levels and qualification rates and the highest rates on aging, immigration (mainly from Latin American countries and the Maghreb) and unemployment [57]. On the contrary, the population with conditions of greater well-being. They got up mainly in the Barcelona district of Sarrià-Sant Gervasi (Southwest of the city) [55]. They concentrate the lowest rates of unemployment, older vehicles, and immigration and the highest levels of income and qualification rates, as well as larger homes [57].

### 2.2. Variables and Information Sources

Many of the data we use come from OpenDataBCN [54]. Open Data BCN, a project that was created in 2010 by implanting the portal in 2011, has been evolving and is now part of Barcelona City Digital’s strategy, the City Council of Barcelona, promoting a plural digital economy. Developing a new model of urban innovation. The Open Data BCN service, managed from the Municipal Data Office, is based on the main international standards and recommendations.

#### 2.2.1. Response Variables 

As response variables, we consider total male and female yearly mortality by neighborhood (crude death rates). Mortality data were observed annually (in the period 2007–2014) at the neighborhood level. Mortality and population data, as well as cartography, were obtained from the OpenDataBCN website of the Barcelona City Council [54].

As explanatory variables, we include socioeconomic indicators and variables related to environmental hazards.

#### 2.2.2. Socioeconomic Indicators

As socioeconomic indicators, we considered disposable household income, the percentage of foreigners from low income countries and housing prices (all by neighborhood) (source in all cases: OpenDataBCN website [54]). 

Disposable household income is, in fact, an index (Barcelona = 100) obtained from OpenDataBCN website [54], and constructed elsewhere [54] from five socioeconomic indicators: (i) unemployment rate (computed as unemployed over resident population aged 16–65 years), (ii) the percentage of resident population (per neighborhood) aged 25 years or more with a university degree, (iii) cars per 1000 over total resident population, (iv) cars more than 16 horsepower (hp) but less than two years old, over the total number of cars less than two years old, and (v) private home resale prices [58].

Given that disposable household income is not likely to capture all the variability contained in socioeconomic indicators, we include some aspects usually related to deprivation such as the percentage of foreigners from low income countries in the neighborhood (according to the 2014 United Nations Development Programme (UNPD)’s human development index [59] stratified by gender. 

With regard to foreigners from low income countries (i.e., immigrants), some studies have shown that they may contribute to increased health inequalities but only in relative terms, compared, for instance, with immigrants from other areas of Spain also with a lower income than the Catalan average (NB: Catalonia is the Autonomous Community to which Barcelona belongs) [60]. 

We also include housing prices in the neighborhood with respect to the average selling prices (€/m^2^) [54]. These prices were estimated as the sale prices of resale properties [61]. In this case, our assumption is that those most deprived neighborhoods, and perhaps also the most polluted, present lower housing prices. 

#### 2.2.3. Air Pollution and Environmental Noise Exposure

Annual average daily levels for the period 2007–2014, of particulate matter (10 micrometres or less in diameter, PM_10_, and 2.5 micrometres or less in diameter, PM_2.5_) nitrogen dioxide (NO_2_), sulphur dioxide (SO_2_), carbon monoxide (CO), benzene and lead, were obtained from the Catalan Government’s Department of Territory and Sustainability website [62]. In the period studied, 13 monitoring stations pertaining to the Catalan Atmospheric Pollution Surveillance and Control Network (XVPCA) were located within the city of Barcelona. In this case, data were collected as point processes located at each of the stations.

As environmental noise data, we included annual average equivalent A-weighted sound pressure levels for daytime (7 h–21 h), evening-time (21 h–23 h) and night-time (23 h–7 h), mapped as isolines, drawn every 5 decibels (db) (A), on the strategic noise map for the ‘Barcelonès I’ agglomeration [63]. This agglomeration includes the cities of Barcelona (with an area of 101.3 km^2^, 62 km^2^ of which corresponds to urban land) and Sant Adrià del Besos (3.87 km^2^ of urban land located on the coast to the north of and surrounded by Barcelona) [64]. Further information can be found in [47]. 

We included land use variables (the source in all cases was the OpenDataBCN website [54]) in an attempt to control for other types of environmental exposures (i.e., other air pollutants measured in a few stations such as nitrogen monoxide—NO-, arsenic, nickel or cadmium; environmental noise not related to traffic, such as the noise resulting from public works or what could be derived from the activities of the port of Barcelona, etc.). We believe that these variables, along with air pollutants and environmental noise, would approximate traffic-related air pollution more efficiently. In particular, we included the percentages of the surface area of the neighborhood intended for public services, industries and infrastructures, roads, urban parks and forest parks. In addition, we also included density of population that, although it is often used as land use variable, it can also be considered as another socioeconomic variable (a less densely populated neighborhood will be more affluent).

### 2.3. Statistical Analysis 

For each sex, we assumed the observed cases of deaths (being a discrete variable, i.e., a counting variable) followed a Poisson distribution,

$$O_{it}\sim Poisson\left(\mu_{it}Pop_{it}\right)$$

where *O_it_* denoted the observed cases of death for a particular sex in the neighborhood *i* (*i* = 1, …, 73) in year *t* (*t* = 2007, …, 2014). *μ_it_* was the relative risk in the neighborhood *i* in year *t*, and *Pop_it_* was the population for a particular gender in the neighborhood *i* in year *t*. 

We are interested in modeling relative risks, which measure the association between the risk factors, and outcome (death in our case). As risk factors we include socioeconomic indicators (disposable household income, percentage of foreigners from low level income countries and housing prices), environmental variables (air pollutants—PM_10_, PM_2.5_, NO_2_, SO_2_, CO, benzene and lead, environmental noise levels and land use variables) and the interaction between air pollutants and environmental noise variables and disposable household income.

The relative risk is associated with risk factors by means of spatio-temporal ecological regression. In our case, this regression was formulated as the following mixed model with two levels: neighborhoods (denoted by *i*) and year (denoted by *t*):

(1) $$\begin{array}{l} \log\left(\mu_{it}\right)=\alpha_{i}+\sum_{q=2}^{5}\beta_{q}HI_{q,it}+\sum_{l=1}^{2}\theta_{1,l}Foreigners_{l,it}+\theta_{2}{housing_{prices}}_{it}\\ \;+\gamma_{1}Pollutant_{it}+\sum_{l=1}^{3}\gamma_{2,l}Noise_{l,it}+\sum_{l=1}^{6}\gamma_{3,l}land\_use_{l,it}\\ \;+\sum_{q=2}^{5}\omega_{1,q}HI_{q,it}:Pollutant_{it}\\ \;+\sum_{l=1}^{3}\left(\sum_{q=2}^{5}w_{2l,q}HI_{q,it}:Noise_{l,it}\right)+\log\left(Pop_{it}\right)+\delta_{1}Pop4564_{it}\\ \;+\delta_{2}Pop65_{it}+S_{i}+T_{t}+\eta_{it} \end{array}$$

where *HI_q,it_* denoted the *q*-th quintile of disposable household income in neighbourhood *i* in year *t* (all the quintiles were constructed for each year separately). The first quintile was taken as a reference value; *Foreigners_l,it_* was the percentage of foreigners from low level income countries in the neighbourhood *i* in year *t* of sex *l* (males, females); *housing_prices_it_* were housing prices in the neighbourhood with respect to the average selling prices in the neighbourhood *i* in year *t*; *Pollutant_it_* denoted the annual average daily level of the pollutant (PM_10_, PM_2.5_, NO_2_, SO_2_, CO, benzene and lead), in neighbourhood *i* and year *t*; *Noise_l,it_* the annual average of environmental noise levels, per neighbourhood, year and for *l*-time (*l* = daytime, evening-time and night-time); *land_use_l,it_* denoted the land use variables (*l* = surface area in the neighbourhood corresponding to public services, industries and infrastructures, roads, urban parks and forest parks, and density of population), with the symbol ‘:’we denoted the interaction between air pollutants and environmental noise variables and quintiles of disposable household income. β’s, γ’s, θ’s and ω’s were unknown parameters.

### 2.4. Mutual Standardization Problem

Rosenbaun and Rubin show that the use of standardized rates as the response variable in ecological regression models leads to biased results if only the dependent variable, and not the predictor, is adjusted for the same confounder, usually age distribution (problem known as the ‘mutual standardization problem’) [65]. Unbiased estimators can be obtained by using crude rates as the response variable (i.e., dependent variable) and entering age (as an average or the age structure) as an (additional) explanatory variable of the model (details can be found in [66]). For this reason, in the specified models we used the crude death rate of the neighbourhood (that is to say, we include population, *Pop_it_*, male or female, of the neighbourhood *i* in year t, as an offset—i.e., denominator), and the age structure of the population (i.e., the percentage of the population aged between 45 and 64 years (both inclusive), *Pop*4564*_it_*, and the population over 65, *Pop*65*_it_*) as additional regressors. To avoid any problems of collinearity with the other two age groups, the first age group (≤44 years) was not included in the model. The coefficients associated with the age structure, δ’s, were also unknown.

### 2.5. Spatio-Temporal Adjustment

Note that we included several random effects (S_i_, α_i_, T_i_, η_it_) as explanatory variables in the model. These, collect unobservable confounders that could also explain the relative risk. 

When one has a spatial design (as in our case), the most important source of non-observed confounding is ‘spatial dependence’ or clustering. That is to say, areas that are close in space show more similar behavior than areas that are not close. In fact, this dependence could be the consequence of unobserved confounders that were spatially distributed (in our case, probably other socioeconomic or environmental variables that have been omitted from the model). To capture the spatial dependency, in the regression we included a structured random effect with a Matérn structure explicitly constructed through the Stochastic Partial Differential Equation approach [67], indexed by the neighborhood (S_i_). Further, by introducing an additional unstructured random effect into the model, indexed by both neighborhood and year (α_i_), we also controlled for the presence of heterogeneity, that is to say, unobserved variables, invariant over time, that are specific to the unit of analysis.

Finally, we controlled for temporal trends, as well as temporal heterogeneity, including a random effect structured as a random walk of order 1 [68], indexed by year (T_i_); and for spatio-temporal interaction including a random effect indexed by both neighborhood and year (η_it_).

### 2.6. Addressing the Misalignment Problem

Note that in our case health data (i.e., the response variables) observed at the ecological level of neighborhoods are misaligned [44] with the two main environmental hazard variables, air pollutants and environmental noise levels. Air pollution levels were collected at point locations, specifically at each one of the air pollution monitoring stations. However, these locations did not coincide with the locations of the response variable (observed at the area level, i.e., neighborhood). Noise levels were recorded using a different spatial resolution (i.e., isolines drawn every 5 dB (A)) to that of the response variables.

The problem of misalignment leads to measurement errors in exposure to both atmospheric and acoustic contamination, errors that are not random but systematic. If these errors are not corrected leads to biased and inconsistent (i.e., asymptotically biased) estimates and erroneous standard errors in the estimates of the parameters. These results in the inference being greatly compromised. Therefore, in order to obtain correct estimates, these measurement errors need to be taken into consideration. In this paper, we use a consistent and efficient fully Bayesian method to address the misalignment issue [47,48,69]. As a result of computational problems, we did not use Markov chain Monte Carlo (MCMC) but rather the Integrated Nested Laplace Approximation (INLA) [70,71] which is a computationally efficient alternative to MCMC. 

As is known, in Bayesian analysis the choice of the prior may have a considerable impact on the results. For this reason, we use penalising complexity (PC) priors here. These priors are invariant to re-parameterisations and have robustness properties [72].

### 2.7. Assessing the Exposure and Susceptibility Differentials

Note that the misalignment problem prevented us from directly assessing the differential exposure. In fact, the levels of air pollutants and environmental noise cannot be assigned, without error, to one or another neighborhood in Barcelona. However, Equation (1) can be used, in addition to directly assessing the differential susceptibility by evaluating the estimates of the parameters ω, to predict the air pollutants and the environmental noise levels in the location of the response variables. 

To assess the exposure differential, we adopt two complementary strategies. First, we use the posterior mean of each environmental hazard variable in quintiles of disposable household income to test whether the samples originated from the same distribution. Given that the distributions of the predicted levels of environmental hazard variables were not symmetrical, we use the Kruskal-Wallis H nonparametric test. However, the results of this test did not inform us about the sign of the relationships, that is, if the most economically deprived neighborhoods had higher levels of environmental hazard variables. For this reason, we estimate a generalized additive model (GAM), foreseeing the possibility of a non-linear relationship. The dependent variable here is the posterior mean of each environmental hazard variable (i.e., air pollutant and environmental noise) and the explanatory variable is the disposable household income. We should point out that we are only interested in the approximate significance of the non-linear smooth slope in the GAM [73] and, in the form of such relationship, if any.

To evaluate the susceptibility differential, we take the predictions of the environmental hazard variables in neighborhoods to build an indicator of a polluted neighborhood. In particular, we consider that a neighborhood is polluted if the (predicted) levels of air pollutants (PM_10_, PM_2.5_, NO_2_, SO_2_, CO, benzene, and lead) and of the environmental noise variables (daytime, evening-time and night-time) were in the fourth or fifth quintiles. In all cases, the quintiles were constructed separately for each year.

Using this indicator, we estimate an additional (summary) Equation:

(2) $$\begin{array}{l} \log\left(\mu_{it}\right)=\alpha_{i}+\sum_{q=2}^{5}\beta_{q}HI_{q,it}+\lambda Polluted\_neighbourhood_{it}\\ \;+\sum_{q=2}^{5}\omega_{q}HI_{q,it}:Polluted\_neighbourhood_{it}+\theta housing\_prices_{it}\\ \;+\sum_{l=1}^{6}\gamma_{l}land\_use_{l,it}+\log\left(Pop_{it}\right)+\delta_{1}Pop4564_{it}+\delta_{2}Pop65_{it}+S_{i}\\ \;+T_{t}+\eta_{it} \end{array}$$

In this case, the parameters of interest are β, λ and, above all, ω’s. These parameters will indicate the presence and the relative importance of the differential susceptibility.

All analyses, conducted separately for men and women [74], were performed with the free software R (version 3.6.1) [75] made available through the INLA package [68,70].

## 3. Results

**Figure 1:**
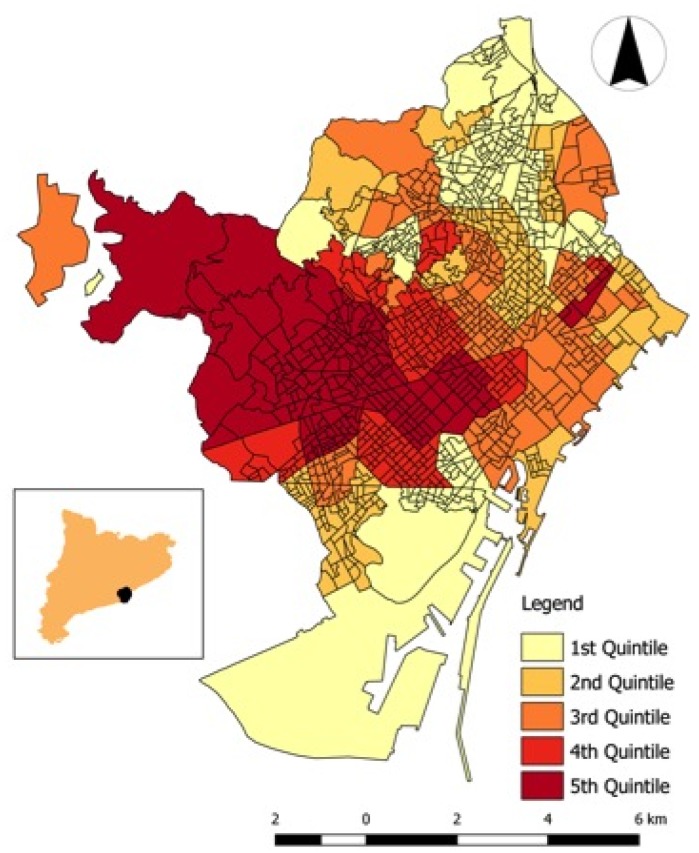
Spatial distribution of the disposable household income by neighbourhoods in Barcelona ^1^. Source: OpenDataBCN website (http://opendata.bcn.cat/opendata/en/cataleg/) [54] and our Own. ^1^ Median of the period 2007–2014.

**Figure 2:**
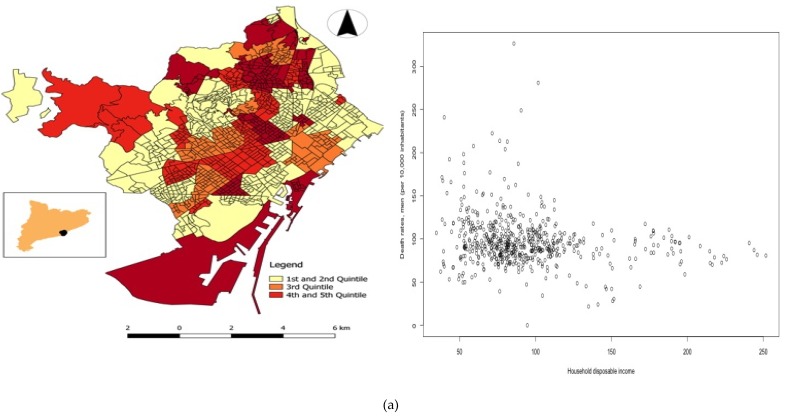

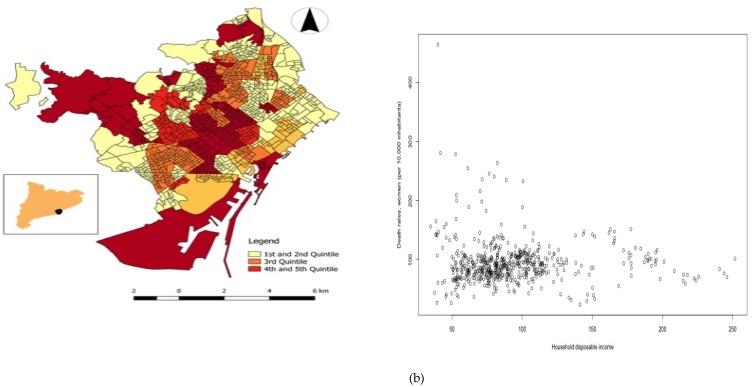
Spatial distribution of the death rates (per 10,000 inhabitants) by neighbourhoods in Barcelona ^1^. (**a**) men, and its relation with disposable household income. (**b**) women, and its relation with disposable household income. Source: OpenDataBCN website (http://opendata.bcn.cat/opendata/en/cataleg/) [54] and our own. ^1^ Median of the period 2007–2014.

In Table 1 and Table 2, and in Figure 1 and Figure 2, we show the descriptive of the variables analysed. There is significant asymmetry in the distribution of all of them (note especially, land use), and some of the variables have an interquartile range which is extremely large when compared to the median (i.e., percentage of the surface of the neighborhood planned for forest and urban parks, and, above all, for industries and infrastructures), and, albeit to a much lesser extent, socioeconomic variables (an interquartile range between 38% and 46% of the median). Note that the variation was not as significant (in relative terms) in the response variables (crude death rates), with an interquartile range about 28–33% of the medians (see Table 1).

The dispersion of the environmental hazard variables was much lower than the rest of explanatory variables, where only benzene had an interquartile range near 100% of its median, while SO_2_, NO_2_, and CO were near 50% of their medians and then the rest had much smaller dispersions (see Table 2). Despite having many observation points in the city, the very low dispersion of environmental noise variables should be noted. 

Moreover, note that while the particles did not exceed the values set in World Health Organization (WHO) air quality guidelines (25 μg/m^3^ daily mean for PM_2.5_—10 μg/m^3^ annual mean- and 50 μg/m^3^ for PM_10_—20 μg/m^3^ annual mean-), NO_2_ exceeded them enough to be noted (40 μg/m^3^ annual mean) (see Table 2). In terms of environmental noise, daytime and evening-time noise exceeded the 55 dB threshold established by the European Union (to reduce ‘annoyance’) and the 50 dB threshold for night-time noise (to reduce sleep disturbance). Furthermore, all of them are well beyond the WHO’s recommended 40 dB threshold. Note that, for all three cases, more of 75% of the observation points exceeded these limits, thus can be considered as having an adverse effect on health [47].

The spatial distribution of disposable household income by neighborhoods (median of the period 2007–2014) is shown in Figure 1. Neighborhoods with a disposable household income located in the upper quartiles (fourth and fifth) were concentrated around an axis with the origin being the city center and one end in the northwest. The spatial distribution for the median of death rates by neighborhoods in Barcelona (2007–2014), was very similar for men and for women (Figure 2a,b). In both cases, there was an (imperfect) axis south-north concentrating the neighborhoods with death rates in the upper quartiles. To better see potential associations, in the same Figure 2, we draw in scatter plots of death rates versus disposable household income for 2007–2014. Although the dispersion was high, it was observed that the neighborhood with the highest disposable household income had the lowest death rates. This association, however, seems to be less pronounced for women.

**Figure 3:**
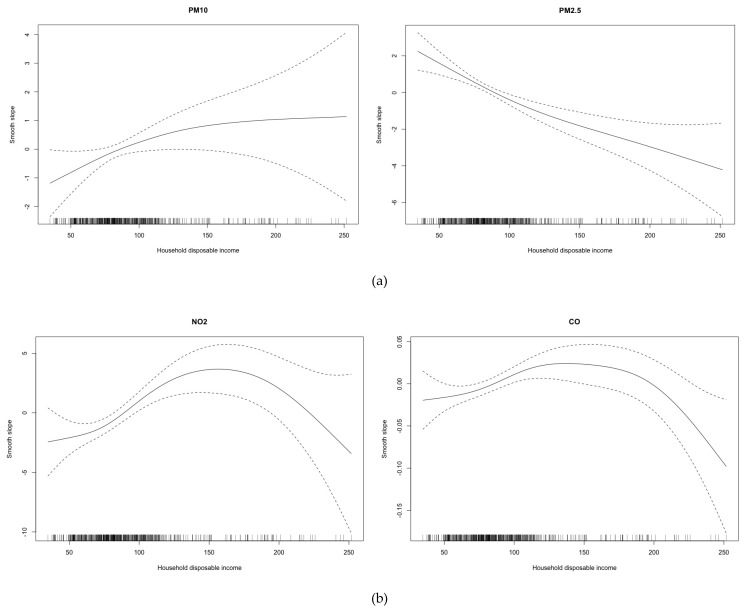

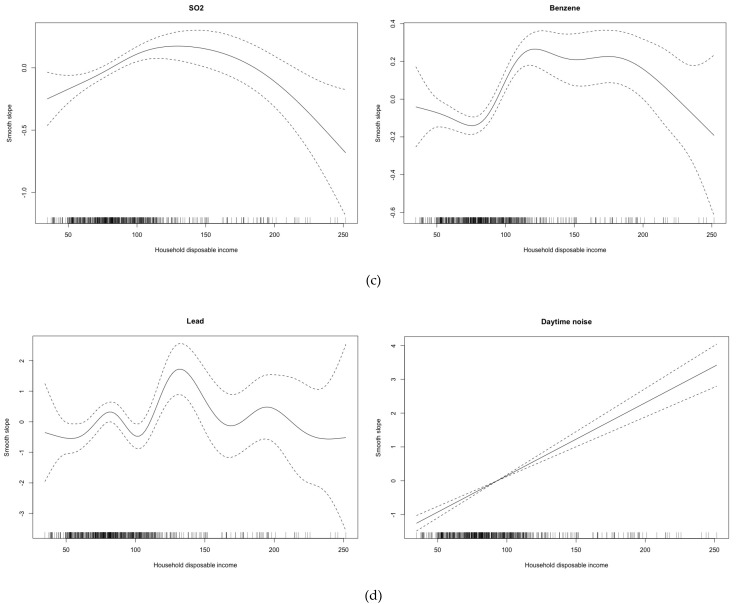

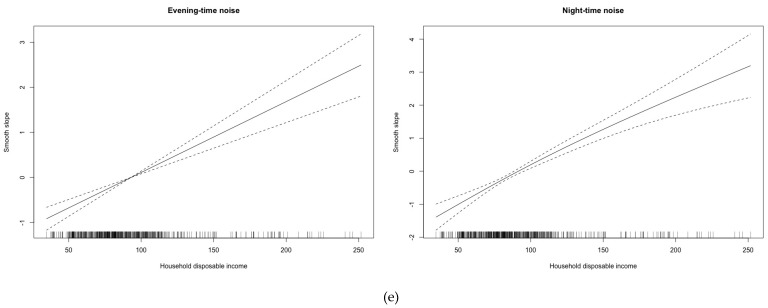
Smoothing of the relationship between the environmental hazard (y-axis) variables and the disposable household income (x-axis), Barcelona, 2007–2014. (**a**) PM_10_ and PM_2.5_; (**b**) NO_2_ and CO; (**c**) SO_2_ and benzene; (**d**) lead and daytime noise; (**e**) evening-time and night-time noise. Approximate significance of the non-linear smooth slope [73], in all cases *p* < 0.01.

In all cases, we could not accept that samples of environmental hazard variables originated from the same distribution (see Table 3). In particular, observing the shape of the curves in Figure 3, it would seem that during 2007–2014, there was a differential exposure, which was higher in the most deprived neighborhoods in the case of PM_2.5_ (Figure 3a), NO_2_ (Figure 3b), benzene (Figure 3c), SO_2_ (Figure 3c) and lead (Figure 3d). In the latter two cases, we should note that the neighborhoods in the fifth quintile of disposable household income were exposed to lower levels of contaminant and the first two quintiles were at the highest levels. Differential exposure was also observed for PM_10_ (Figure 3a) and CO (Figure 3b), although in these cases the neighborhoods in the last two quintiles of disposable household income (i.e., the most affluent) were those who were exposed to higher levels of these two pollutants. This was much more evident in the case of environmental noise (Figure 3d,e).

Table 4 depicts the results for differential susceptibility. For both men and women, the risk of dying due to environmental hazards in a very affluent neighborhood (located on the fifth quintile of disposable household income) is about 30% lower than in a very depressed neighborhood (located in the first quintile). Note that there is no difference in the risk of dying from pollution in neighborhoods located in the second quintile (i.e., the interaction was not statistically significant). 

With individual air pollutants, the behavior for men and women appears to be different (except for benzene) (see Table 4). For men, the risk of dying from CO, benzene, NO_2_ and/or SO_2_ pollution (in decreasing order) is lower in the most affluent neighborhoods (located in the fifth quintile of disposable household income). For women, the risk of dying because of benzene pollution is lower in the most affluent neighborhoods and higher for those neighborhoods located in the second quintile in the case of PM_2.5_. In the case of environmental noise, for both men and women, the risk of dying due to evening-time noise was higher in the most affluent neighborhoods (i.e., fifth quintile) and lower in the neighborhoods located in the second quintile. In the case of night-time noise, the risk of dying is lower for the most affluent neighborhoods, albeit only for men. In the case of daytime noise, there are no differences in the risk by quintiles for disposable household income.

The main effects of the explanatory variables of interest were to be expected. There is a 25% risk (for men) of dying in a neighborhood with serious environmental hazard problems. This is 40% higher than in a neighborhood without such problems. Note, however, that not all air pollutants and all environmental noise variables have an associated increased risk of dying (for instance, benzene for both genders and PM_10_ for men, or evening-time and daytime noise). For disposable household income, the higher the income quintile the neighborhood is in, the lower the risk of dying is. 

## 4. Discussion

In summary, we have found evidence of differential susceptibility in that the effect of environmental hazards was more harmful to the residents of Barcelona’s most deprived neighborhoods (see Appendix A). Our results are consistent with those found in most studies in Europe, as well as in some non-European studies, about the existence of a differential susceptibility [7].

However, it appears that this increased susceptibility cannot be attributed to a single problem but rather to a set of environmental hazards that, overall, a neighborhood may present. In fact, only in the case of benzene would there be systematic behavior. At any rate, both men and women living in the neighborhoods located in the fifth quintile of disposable household income, presented a lower risk of dying (statistically significant) than those inhabitants of neighborhoods located in the first quintile. 

In terms of the other environmental hazards, first, the relative risks for the fifth quintile of disposable household income for some other air pollutants (i.e., NO_2_, CO and SO_2_) were only statistically significant for men. Second, there appears to be no differential susceptibility in the case of particles (at least when taken individually). Finally, in the case of environmental noise and evening noise in particular, it seems that there was an inverse differential susceptibility, that is to say, the relative risks in the upper quintile were higher than the risks in the more deprived neighborhoods.

We believe that this heterogeneity in differential susceptibility to individual environmental hazards, except perhaps in the case of benzene, is largely related to the heterogeneity of the differential exposure to environmental hazards, also taken individually (as shown in Figure 3). In fact, except in the case of benzene, we would not venture to state that we have found a differential exposure to environmental problems taken individually. In this sense, our results are in line with those obtained by other European studies that analyze air pollutants (above all) individually. That is to say, we also find mixed results when we assess environmental problems individually, unlike most non-European studies, especially in North America, which find a differential exposure (higher in areas with low-socioeconomic status) to the air pollutants criteria [12].

These discrepancies between the finding of a differential susceptibility and the finding of a set of environmental problems that a neighborhood may suffer, along with the mixed results of differential susceptibility to individual environmental hazards, could be explained by our most serious limitations. We have used an ecological observational research design. By being observational, this could mean there are unobserved, and therefore uncontrolled, confounders that may contribute to a differential exposure beyond environmental hazards which, in turn, might explain why there are modifiers of their effects [7]. Being ecological, greater environmental problems in a neighborhood do not necessarily mean greater exposure for all its inhabitants. However, in our study, we have controlled for unobserved confounding (both spatially or temporally structured as well as unstructured) and we have corrected other methodological problems associated with exposure, such as spatial misalignment. 

For all these reasons, we venture to conclude that the inequalities of health hazards are hidden in the air we breathe and the noise we exposed to. Air and noise quality depend on where we live and our day-to-day environment. Both are related to some socioeconomic factors, of which some of the major issues are the cost of housing and the kind of job we have and work we do. These may well reinforce potential negative health impacts because of people needing to have greater mobility, needing to use their vehicles to get from A to B quickly to avoid losing valuable working/productive time, all the while increasing traffic congestion and pollution levels. In these cases, deprivation is usually found in the social determinants (e.g., distance to work, type of work, time to rest, banlieues etc.). While rural areas may (for the moment) show a different spectrum, at present the general trend is towards major urbanization (particularly in Less Developed Countries (LDCs)) and to more specialized zoning with concentrated malls and shopping centers on the city outskirts. Again, this disregards jobs or goods and services within walking distance, in favor of the car, and so often decreases physical activities. 

City-level decision-makers usually neglect the former negative externalities likely due to the socially unequal negative impacts. Today better zoning and greater concern for healthier lifestyles are changing old perspectives which, in turn, may even enhance a city’s attractiveness and achieve greater social cohesion by reducing health inequalities and segregation. Out of genetics and the proper healthcare access, the search for a better environment is a rather endogenous health policy with a higher impact than spending on health and social services. This is particularly important in LDCs, although the heterogeneity observed in urban areas of Developed Countries (e.g., in the neighborhoods in Barcelona) also justify greater concern about the noise levels we are exposed to and the quality of the air we breathe. Differential exposure and incidence by income groups show the spatial nature of the problem and analysis of such offers some clues for more evidence-based environmental public health policies.

## 5. Conclusions

The effect of environmental hazards was more harmful to the residents of Barcelona’s most deprived neighborhoods; that is to say, we have found evidence of the existence of a differential susceptibility to exposure. This increased susceptibility cannot be attributed to a single problem but rather to a set of environmental hazards that, overall, a neighborhood may present. On the contrary, we would not venture to state that we have found a differential exposure to environmental problems, at least taken individually. It is very likely that this discrepancy may be due to the use of an ecological observational research design. 

## Figures and Tables

**Table 1:** Descriptive statistics. Neighborhoods in Barcelona, 2007–2014.

Variable	Mean	Standard Deviation	Median	First Quartile	Third Quartile	Minimum	Maximum
Death rates (per 10,000 inhabitants)							
Males	97.62	30.31	93.33	81.60	107.96	0.00	326.53
Females	94.18	36.50	89.40	74.98	104.39	23.70	464.44
Disposable household income (Barcelona = 100)	93.03	37.62	84.45	70.05	104.80	34.70	251.70
Foreigners from low-income countries (%)							
Males	5.65	2.50	4.96	4.11	6.41	1.62	21.95
Females	4.86	1.70	4.52	3.84	5.56	1.84	18.97
Housing prices ^1^ (€/m^2^)	3271.99	931.76	3174.00	2603.00	3809.00	1360.00	6298.00
Land use variables ^2^							
Public services (%) ^3^	11.12	7.64	8.93	6.31	12.81	2.72	49.85
Industries and infrastructures (%) ^3^	5.03	11.80	0.27	0.00	3.18	0.00	70.21
Roads (%) ^3^	27.36	8.00	28.93	21.85	33.77	5.43	39.13
Urban parks (%) ^3^	14.93	10.96	12.26	7.13	20.49	0.97	47.75
Forest parks (%) ^3^	6.29	17.27	0.00	0.00	0.00	00.00	82.60
Density of population (inhabitants/km^2^)	24,819.30	15,220.62	24,299.20	11,628.32	35,175.88	70.38	60,026.83

73 neighbourhoods. ^1^ Sale prices of resale properties on sale. ^2^ Residential area not included. ^3^ Percentages of the surface area of the neighborhood.

**Table 2:** Descriptive statistics. Environmental hazard variables ^1^. Barcelona, 2007–2014.

	Air Pollutants ^2^							Environmental Noise ^3^		
	PM_10_^4^	PM_2.5_^4^	NO_2_^4^	SO_2_^4^	CO ^5^	Benzene ^4^	Lead ^6^	Daytime ^7^	Evening-Time ^7^	Night-Time ^7^
N ^8^	11	9	8	8	8	6	9	16,742	16,742	16,742
Mean	32.50	17.11	44.42	2.80	0.41	1.74	13.20	62.12	60.58	54.57
Standard deviation	8.693	2.855	11.473	0.992	0.1285	0.889	3.194	7.518	7.466	8.028
Minimum	19.00	12.00	27.00	1.00	0.20	0.70	10.30	0.00	0.00	0.00
Maximum	62.00	24.00	74.00	5.00	0.70	3.40	32.00	79.00	77.00	73.00
Percentiles										
25 (1st quartile)	26.25	15.00	36.00	2.00	0.300	1.08	11.23	58.00	57.00	50.00
50 (median)	31.00	17.00	42.00	3.00	0.400	1.40	12.30	63.00	62.00	56.00
75 (3rd quartile)	36.75	19.00	51.75	3.75	0.500	2.73	14.03	67.00	66.00	60.25

^1^ Original data. ^2^ Annual average daily levels. ^3^ Annual average equivalent A-weighted sound pressure levels. ^4^ μg/m^3^; ^5^ mg/m^3^; ^6^ ng/m^3^. ^7^ dB. ^8^ Number of monitoring stations for air pollutants; Number of observation points for environmental noise.

**Table 3:** Assessment of the exposure differential ^1^. Neighbourhoods in Barcelona, 2007–2014.

Environmental Hazard Variables	Quintiles of Disposable Household Income (Barcelona = 100)					Kruskal-Wallis H ^3^
	1st Quintile	2nd Quintile	3rd Quintile	4th Quintile	5th Quintile	*p*-Value
	34.70–64.90	64.90–79.32	79.32–92.30	92.30–110.76	110.76–251.70	
Air pollutants ^2^						
PM_10_	35.00 (0.085) [35.03]	34.98 (0.111) [34.97]	34.97 (0.123) [34.92]	34.92 (0.105) [34.91]	35.02 (0.194) [34.98]	<0.001
PM_2.5_	17.41 (0.193) [17.50]	17.34 (0.218) [17.45]	17.34 (0.169) [17.40]	17.33 (0.118) [17.34]	17.22 (0.121) [17.20]	<0.001
NO_2_	48.38 (0.568) [48.61]	48.15 (0.505) [48.37]	48.16 (0.330) [48.24]	48.13 (0.325) [48.13]	48.09 (0.309) [48.12]	<0.001
SO_2_	4.14 (3.828) [2.77]	3.87 (3.350) [2.83]	3.62 (2.796) [2.57]	5.05 (3.349) [4.61]	3.89 (2.656) [3.64]	0.006
CO	0.42 (0.024) [0.41]	0.44 (0.032) [0.42]	0.44 (0.030) [0.43]	0.44 (0.023) [0.43]	0.45 (0.027) [0.44]	<0.001
Benzene	2.58 (0.564) [2.71]	2.43 (0.427) [2.56]	2.35 (0.425) [2.38]	2.17 (0.498) [2.23]	2.29 (0.534) [2.45]	<0.001
Lead	13.38 (0.169) [13.31]	13.42 (0.217) [13.34]	13.37 (0.177) [13.51]	13.42 (0.162) [13.40]	13.32 (0.194) [13.34]	0.001
Environmental noise ^2^						
Daytime	63.33 (1.731) [63.56]	64.07 (1.559) [63.75]	63.84 (1.786) [63.65]	64.86 (1.863) [65.45]	65.71 (1.951) [66.07]	<0.001
Evening-time	61.08 (1.852) [61.34]	61.57 (1.693) [61.94]	61.23 (1.978) [61.32]	62.33 (2.092) [62.33]	62.74 (2.101) [63.29]	<0.001
Night-time	54.52 (2.148) [54.55]	55.42 (1.915) [54.94]	55.13 (2.130) [55.15]	56.29 (2.160) [56.37]	57.01 (2.239) [57.62]	<0.001

^1^ Prediction of air pollutants and environment noise levels on health data locations (centroid of neighbourhoods). ^2^ Mean (Standard deviation) [Median]. ^3^ Non-parametric Kruskal-Wallis H test for testing whether samples originated from the same distribution.

**Table 4:** Assessment of the susceptibility differential. Neighborhoods in Barcelona, 2007–2014. Relative risks (95% credibility intervals).

Title	Male	Female
Polluted Neighbourhood [Non-polluted]	1.249 (1.019–1.526)	1.399 (1.087–1.797)
Disposable household income [1st Quintile]		
2nd Quintile	0.972 (0.929–1.017)	0.987 (0.941–1.035)
3rd Quintile	0.947 (0.895–0.999)	0.953 (0.899–0.999)
4th Quintile	0.957 (0.902–1.015)	0.938 (0.882–0.997)
5th Quintile	0.924 (0.854–0.999)	0.913 (0.843–0.989)
Interactions with Polluted Neighbourhood		
2nd Quintile	1.017 (0.899–1.152)	0.973 (0.850–1.114)
3rd Quintile	0.823 (0.653–1.041)	0.716 (0.539–1.042)
4th Quintile	0.857 (0.684–1.077)	0.711 (0.545–1.045)
5th Quintile	0.794 (0.632–1.002)	0.706 (0.537–0.932)
Air pollutants		
PM_10_	**1.377 (0.907–2.081)**	
Benzene	**1.077 (1.004–1.207)**	**1.117 (1.018–1.240)**
Environmental noise		
Daytime	**1.219 (0.904–1.580)**	**1.044 (0.748–1.457)**
Evening-time	**1.131 (1.014–1.261)**	**1.117 (0.954–1.309)**
Interactions with quintiles of income		
Air pollutants [1st Quintile]		
PM_2.5_-2nd Quintile		**1.253 (0.956–1.640)**
NO_2_-5th Quintile	**0.918 (0.795–1.059)**	
CO-5th Quintile	**0.143 (0.007–2.835)**	
SO_2_-5th Quintile	**0.987 (0.972–1.003)**	
Benzene-5th Quintile	**0.850 (0.704–0.989)**	**0.898 (0.705–0.993)**
Environmental noise [1st Quintile]		
Evening-time noise		
2nd Quintile	**0.944 (0.887–0.999)**	**0.950 (0.874–1.033)**
5th Quintile	**1.125 (1.008–1.269)**	**1.010 (0.871–1.148)**
Night-time noise-5th Quintile	**0.840 (0.668–1.055)**	

[Reference category between brackets]. The 95% credibility interval did not contain the unity; the 90% credibility interval did not contain the unity. Models adjusted for housing prices and land use variables (percentages of the surface area of the neighborhood on public services, industries and infrastructures, roads, urban parks, forest parks and density of population), with spatio-temporal adjustment. The bold indicates that only relative risks whose credibility interval 90% or 95% did not contain the unit.

## Abbreviations

(A)	A-weighted
db	Decibels
CO	Carbon monoxide
GAM	Generalized Additive Models
IDESCAT	Statistical Institute of Catalonia
INLA	Integrated Nested Laplace Approximation
MCMC	Markov Chain Monte Carlo
NO_2_	Nitrogen dioxide
PC-priors	Penalising complexity priors
PM_2.5_	2.5 micrometres diameter particulate matter
PM_10_	10 micrometres diameter particulate matter
SO_2_	Sulphur dioxide
SPDE	Stochastic partial differential equations
UNPD	United Nations Development Programme
WHO	World Health Organisation
XVPCA	Catalan Atmospheric Pollution Surveillance and Control Network

## Appendix A

### Key Messages

We have found evidence of differential susceptibility in that the effect of environmental hazards was more harmful to the residents of Barcelona’s most deprived neighbourhoods.

It appears that this increased susceptibility cannot be attributed to a single problem but rather to a set of environmental hazards that, overall, a neighborhood may present.

The heterogeneity in differential susceptibility to individual environmental hazards, except perhaps in the case of benzene, is largely related to the heterogeneity of the differential exposure to environmental hazards, also taken individually.

These discrepancies between the finding of a differential susceptibility and the finding of a set of environmental problems that a neighborhood may suffer, along with the mixed results of differential susceptibility to individual environmental hazards, could be explained by the use of an ecological observational research design.
